# Endophilin A2-mediated alleviation of endoplasmic reticulum stress-induced cardiac injury involves the suppression of ERO1α/IP_3_R signaling pathway

**DOI:** 10.7150/ijbs.60110

**Published:** 2021-08-26

**Authors:** Yun Liu, Ruixiang Hu, Huanjia Shen, Qinxin Mo, Xinqiuyue Wang, Guiping Zhang, Sujuan Li, Guanfeng Liang, Ning Hou, Jiandong Luo

**Affiliations:** 1 The Fifth Affiliated Hospital, Key Laboratory of Molecular Target & Clinical Pharmacology and the State & NMPA Key Laboratory of Respiratory Disease, School of Pharmaceutical Sciences, Guangzhou Medical University, Guangzhou, 511436, P.R. China.; 2 Department of Gastrointestinal Surgery, First Affiliated Hospital of Jinan University, Guangzhou 510630, P.R. China.; 3 Guangzhou Institute of Cardiovascular Disease, Guangzhou Key Laboratory of Cardiovascular Disease, and the Second Affiliated Hospital, Guangzhou Medical University, Guangzhou 510260, P.R. China.

**Keywords:** endophilin A2, cardiac injury, ER stress, ERO1α, IP_3_R

## Abstract

Cardiac injury upon myocardial infarction (MI) is the leading cause of heart failure. The present study aims to investigate the role of EndoA2 in ischemia-induced cardiomyocyte apoptosis and cardiac injury. *In vivo*, we established an MI mouse model by ligating the left anterior descending (LAD) coronary artery, and intramyocardial injection of adenoviral EndoA2 (Ad-EndoA2) was used to overexpress EndoA2. *In vitro*, we used the siRNA and Ad-EndoA2 transfection strategies. Here, we reported that EndoA2 expression was remarkably elevated in the infarct border zone of MI mouse hearts and neonatal rat cardiomyocytes (NRCMs) stimulated with oxygen and glucose deprivation (OGD) which mimicked ischemia. We showed that intramyocardial injection of Ad-EndoA2 attenuated cardiomyocyte apoptosis and reduced endoplasmic reticulum (ER) stress in response to MI injury. Using siRNA for knockdown and Ad-EndoA2 for overexpression, we validated that knockdown of EndoA2 in NRCMs exacerbated OGD-induced NRCM apoptosis, whereas overexpression of EndoA2 attenuates OGD-induced cardiomyocyte apoptosis. Mechanistically, knockdown of EndoA2 activated ER stress response, which increases ER oxidoreductase 1α (ERO1α) and inositol 1, 4, 5-trisphosphate receptor (IP_3_R) activity, thus led to increased intracellular Ca^2+^ accumulation, followed by elevated calcineurin activity and nuclear factor of activated T-cells (NFAT) dephosphorylation. Pretreatment with the IP_3_R inhibitor 2-Aminoethoxydiphenylborate (2-APB) attenuated intracellular Ca^2+^ accumulation, and pretreatment with the Ca^2+^ chelator 1,2-bis(o-aminophenoxy)ethane-N,N,N′,N′-tetraacetic acid (BAPTA) or the calcineurin inhibitor Cyclosporin A (CsA) inhibited EndoA2-knockdown-induced NRCM apoptosis. Overexpression of EndoA2 led to the opposite effects by suppressing ER-stress-mediated ERO1α/IP_3_R signaling pathway. This study demonstrated that EndoA2 protected cardiac function in response to MI via attenuating ER-stress-mediated ERO1α/IP_3_R signaling pathway. Targeting EndoA2 is a potential therapeutic strategy for the prevention of postinfarction-induced cardiac injury and heart failure.

## Introduction

Heart failure is one of the major causes of death worldwide and is also a rapidly growing public health burden that affects approximately 40 million individuals globally^1^. Heart failure develops as a result of the health problems that are related to congenital or acquired conditions, and the major risk factors for heart failure are hypertension, diabetes, atrial fibrillation and ischemic heart disease (IHD). IHD, as a consequence of myocardial infarction (MI), is the most common cause of heart failure 1, 2. The decline in immediate mortality after MI has been well documented and is partially related to the use of more effective treatments, including angiotensin converting enzyme inhibitors, β-blockers, coronary revascularization, implantable cardioverter-defibrillators, and cardiac resynchronization therapeutic strategies 2, 3. However, a history of acute MI is associated with a 5-fold increase in the incidence of heart failure after 5 years of MI 4, 5. Therefore, great interest lies in the discovery of new therapeutic strategies to alleviate adverse cardiac injury and promote cardiac repair.

Accumulating evidence suggests that the regulatory networks involved in cardiac injury is complex; however, cardiomyocyte apoptosis is a crucial factor because massive numbers of cardiomyocytes are lost 6. Ca^2+^ is a ubiquitous intracellular secondary messenger that is involved in apoptosis. Ca^2+^ homeostasis is pivotal for cell survival, and the disruption of intracellular Ca^2+^ homeostasis triggers apoptosis 7, 8. Therefore, one of the strategies aiming to prevent and treat postinfarction-induced cardiac injury focuses on the regulation of Ca^2+^ homeostasis. Inositol 1, -4, -5-trisphosphate receptor (IP_3_R), a ligand-gated Ca^2+^ channel, is primarily localized in the endoplasmic reticulum (ER) membrane. The IP_3_R-mediated Ca^2+^ efflux from the ER regulates a wide range of biological processes, including proliferation, differentiation, metabolism and apoptosis 9, 10. IP_3_R-mediated Ca^2+^ release from the ER regulates mesodermal specification via calcineurin/NFATc3/Etv2 signaling pathway, which is crucial in hematopoietic and cardiac fate divergence 11. Bcl-2, an anti-apoptotic protein, inhibits the IP_3_R-mediated Ca^2+^ release by interacting with IP_3_R and is dedicated to blocking proapoptotic Ca^2+^ signal 12. In another study, receptor-interacting protein 140 (RIP140) was shown to attenuate the IP_3_R-mediated Ca^2+^ release by binding to IP_3_R, thus protecting against neuronal death 13. These studies confirm that IP_3_R activity is vital for Ca^2+^ homoeostasis and cell survival.

Endophilin, which contains a proline-rich peptide ligand of the SH3 domain, has a well-documented role in endocytosis. Endophilin contains two subtypes: endophilin A and endophilin B. Endophilin A2 (EndoA2) is a subtype of the endophilin A family and is a multifunctional protein. Numerous studies suggest that EndoA2 is involved in tumor metastasis 14, atherosclerosis 15, virion production 16 and neuroregulation 17. Our previous study suggested that EndoA2 significantly inhibits rat basilar artery smooth muscle cell apoptosis by directly binding to Bax and preventing Bax translocation from cytoplasm to mitochondria 18. This observation has made a significant contribution to our understanding of the role of endophilin family in cardiovascular diseases, which has never been examined before. Our following-up studies on EndoA2 further confirmed the critical role of EndoA2 in heart diseases 19-21. However, the role of EndoA2 in MI remains poorly understood.

Early in 2003, Chen et al. reported that EndoA2 directly interacted with voltage-gated Ca^2+^ channels in a Ca^2+^-dependent manner and regulated clathrin-mediated synaptic vesicle endocytosis, which implied the association between EndoA2 and Ca^2+^ 17. Based on a previous study showing that EndoA2 interacts with Ca^2+^ channels and another suggesting the ER oxidoreductase 1α (ERO1α)-mediated activation of IP_3_R, we hypothesized that EndoA2 protected the heart against ischemic injury by suppressing the ERO1α-mediated stimulation of IP_3_R activity. In this study, we report that intramyocardial injection of adenoviral EndoA2 (Ad-EndoA2) to overexpress EndoA2 in the murine hearts reduces MI-induced cardiac injury and preserves cardiac function. Cardiac protection by EndoA2 is further confirmed by an *in vitro* study of a cellular model of oxygen and glucose deprivation (OGD). We show that EndoA2 regulates the ER stress/ERO1α/IP_3_R signaling pathway and attenuates ER-stress-induced apoptosis. Our study confirms the contribution of EndoA2 to protect cardiac function upon ischemia-induced injury and underscores the therapeutic potential of EndoA2 to treat heart failure.

## Materials and methods

### Animals

Neonatal Sprague-Dawley rats (1 to 3 days old) and adult male C57BL/6 mice (body weight between 22 to 25 g, aging between 6 to 8 weeks old) were obtained from Guangzhou University of Chinese Medicine (Guangzhou, China) and housed in animal facility with 12/12-h light/dark cycle at a temperature of 25 ± 2 °C and humidity of 60 ± 5 %. The animal protocols of this study were approved by the Institutional Animal Care and Use Committee (IACUC) of Guangzhou Medical University (Guangzhou, China) and all animal studies were performed in accordance with relevant guidelines from the Directive 2010/63/EU of the European Parliament on the protection of animals used for scientific purposes.

### Animal model, echocardiography, and morphometric measures

Male C57BL/6 mice used in this study were randomly divided into each group. Myocardial infarction was generated in the male C57/B6 mice by ligating left anterior descending (LAD) coronary artery as previously described 22. Briefly, mice were anesthetized with sodium pentobarbital (50 mg/kg) intraperitoneally and artificially ventilated with a respirator, and all efforts were made to minimize suffering. MI was followed by making a slipknot (8-0 silk suture) at 2 mm below the border between left atrium and ventricle. Marked color change of the ischemic area and electrocardiograph change were monitored as an indication of successful coronary artery occlusion. The sham-operated mice for control underwent the same experimental procedures as the MI group but without ligation of LAD. A total 25 μL Ad-lacZ or Ad-EndoA2 (10^9^ particles, Shanghai Sunbio Biomedical Technology, Shanghai, CN) was transduced into 3 sites around the infarcted area (anterior wall, lateral wall, and apex area) by intramyocardial injection. When injecting, the needle is within the ventricle muscular wall but not ventricular cavity. Transthoracic echocardiography was performed with a VisualSonics (Vevo 2100, VisualSonics Inc., Ontario, Canada) equipped with a 30 MHz imaging transducer on day 14 after MI. After that, the mice were sacrificed. The hearts were rapidly removed, and the infarct border zone was carefully trimmed. For morphometric measures, hearts were fixed in 4% paraformaldehyde (pH7.4) overnight, and then embedded in paraffin wax. Histological cross sections (5 μm thick) of the hearts were stained with hematoxylin and eosin (H&E). Dead mice were excluded and all animal experiments were performed by an observer blinded to the sample identity.

### 2, 3, 5-triphenyl tetrazolium chloride (TTC) staining

After determination of the cardiac function on day 14, mice were anaesthetized with sodium pentobarbital (100 mg/kg) and assessed to be fully anaesthetized and sacrificed. The ventricles were collected and sliced transversely into 2 mm thick slices. The slices were incubated in 1% TTC (pH 7.4) for 20 min at 37 °C. The infarct area was shown as the area unstained by TTC.

### Masson's trichrome staining

After determination of the cardiac function on day 14, mice were anaesthetized and sacrificed as described above. The hearts were perfused, sectioned and then fixed in paraffin and subjected to Masson's trichrome-staining as we have described previously 23.

### Primary culture of neonatal rat cardiomyocytes (NRCMs)

Neonatal Sprague-Dawley rats (1 to 3 days old) were exposed to hypothermia until they became unresponsive and were then euthanized by cervical dislocation. Primary culture of NRCMs was performed according to previously published method 24. The cells were seeded at a density of 5 x 10^6^ cells/ml in Dulbecco's modified Eagle's medium supplemented with 10% fetal bovine serum and 0.1 mM 5-bromodeoxyuridine. After 24 h, NRCMs were subjected to various treatments as described below. To assess the effects of EndoA2 on cardiac injury, the cells were transfected with EndoA2 siRNA or Ad-EndoA2. After transfection for 36 h, the cells were stimulated with oxygen and glucose deprivation (OGD) for another 12 h. Incubation of sodium phenylbutyrate (4-PBA, Sigma, Boston, MA), Cyclosporin A (CsA, Selleck, Houston, TX), 1,2-bis(o-aminophenoxy)ethane-N,N,N′,N′-tetraacetic acid (BAPTA, Selleck, Houston, TX) or 2-Aminoethoxydiphenylborate (2-APB, Selleck, Houston, TX) was 2 h before OGD stimulation. Cardiomyocytes were cultured in serum-free, glucose and sodium pyruvate- free DMEM (Sigma, USA) at 37 °C in an anoxia chamber (InVivo 500, Skinn Life Science) saturated with 94% N_2_/5% CO_2_/ 1% O_2_.

### Transfection with EndoA2 siRNA or Ad-EndoA2

EndoA2 siRNA transfection was performed as we have previously described 18. The siRNA duplexes against the rat EndoA2 gene were transiently transfected with Hyperfect Transfection Regent. According to our previous studies, we chose to transfect with 20 nM EndoA2 siRNA for 48 h in this experiment. After 48 h, the cells were used for subsequent experiments. The siRNA sequences against ERO1α were generated by Qiagen (Valencia, CA, USA). The target sequences were 5'-CAGCTCTTCACTGGGAATAA-3' as referred to by a reported study 25. The EndoA2 adenovirus was constructed by Shanghai Sunbio Biomedical Technology (Shanghai, CN). On the day of infection, the cells were 50%-60% confluent, then, the cells were infected with Ad-EndoA2 or Ad-lacZ at a multiplicity of infection (MOI) of 50. After 6 h incubation at 37 °C, the transfection solution was replaced with 10% serum-containing medium and incubated for an additional 48 h.

### CCK-8 assay

Cell number was measured by CCK-8 (Dojindo Molecular Technologies, MD, Japan). Cardiomyocytes were plated on 96-well plates at a density of 1-2 x 10^4^ cells for 24 h and then stimulated with OGD for 12 h.10 μl CCK-8 solution was added into each well for another 4 h, and absorbance at 450 nm was measured by microplate reader (Bio-Tek, Winooski, VT, USA).

### Apoptosis detection

Cardiomyocyte apoptosis was detected by Annexin V-FITC Apoptosis Detection Kit (KeyGEN Biotech, Nanjing, China). Cardiomyocytes were collected at a density of 1 × 10^6^ cells/ml, washed with ice-cold PBS twice, centrifuged at 1000 rpm/min for 5 min, and then were resuspended with 1 × binding buffer, mixed with 5 μl Annexin V-FITC and 5 μl PI in darkness at room temperature for 10 min. Then the samples were analyzed by flow cytometer (Beckman Coulter, Fullerton, CA, USA) in 30 min.

### TUNEL staining

Cardiomyocytes and heart tissues were stained with terminal deoxynucleotidyl transferase dUTP nick-end labeling (TUNEL) staining. Fluorescence staining was conducted using *in situ* Cell Death Detection Kit from Roche (Shire Park, UK) according to the manufacturer's instructions. The apoptotic cells were observed under light microscope and the results were expressed as the average number of TUNEL-positive staining cells per 10× magnification field. Cardiomyocytes were stained with α-sarcomeric actinin.

### Western blotting

Protein of cardiomyocytes or heart tissues was lysed in lysis buffer (Beyotime Biotechnology, Shanghai, CN). Protein concentrations were determined by BCA protein assay kit (Beyotime Biotechnology, Shanghai, CN). The SDS-PAGE was used to separate the samples and then transferred the samples onto the PVDF membrane. The membrane was blocked with TBST containing 5% non-fat dry milk at room temperature for 2 h, then washed with TBST for 3 times, followed by incubating the membrane with the specific primary antibody against cleaved caspase-3 (Cell Signaling, Boston, MA, 1:1000, Cat. No.: 9661), CHOP (Cell Signaling, Boston, MA, 1:1000, Cat. No.:2895), IP_3_R (Abcam, MA, USA, 1:1000, Cat. No.: ab108517), ERO1α (Novus, USA, 1:1000, Cat. No.: NB100-2525), EndoA2 (Santa Cruz Biotechnology, Dallas, USA, 1:200, Cat. No.: sc365704), Bcl-2 (Proteintech, Chicago, USA, 1:1000, Cat. No.: 26593-1-AP), Bax (Proteintech, Chicago, USA, 1:1000, Cat. No.: 50599-2-lg), GRP78 (Proteintech, Chicago, USA, 1:1000, Cat. No.: 11587-1-AP) at 4 °C overnight. The next day, the membranes were incubated with secondary antibody (Cell Signaling, Boston, MA) conjugated to horseradish peroxidase for 1 h. Blots were developed using a Immobilon Western Chemiluminescent HRP Substrate kit (Millipore) and molecular band intensity was determined by densitometry with Image J program (NIH, Maryland, USA).

### Immunofluorescence (IF) staining

Myocardium tissues or cultured NRCMs were washed three times with PBS, followed by being fixed with 4% paraformaldehyde for 30 min, permeabilized with 0.01% Triton X-100 for 30 min, and blocked with goat serum for 1 h. Then the cells were incubated with anti-sarcomeric actinin (1: 500, Cell Signaling, Boston, MA), anti-EndoA2 (1:200, Santa Cruz Biotechnology, Dallas, USA), p-IP_3_R (1:200, Abcam, MA, USA) in a humidified chamber at 4 °C overnight. After being washed for three times with PBS, the cells were then incubated with a goat anti-mouse IgG (H+L) secondary antibody (1:1000, Cell Signaling, Boston, MA) or goat anti-rabbit IgG (H+L) secondary antibody (1:1000, Cell Signaling, Boston, MA) for 1.5 h. Finally, the nuclei were counterstained with DAPI and the cells were observed by a laser scanning confocal microscope (Nikon, Tokyo, Japan).

### Cellular Ca^2+^ measurement

The fluorescent calcium indicator Fluo-4 AM (2.5 µM, Beyotime Biotechnology, Shanghai, CN) was used to measure intracellular free Ca^2+^ level. The cells were monitored using Nikon A1 laser scanning confocal microscope (Nikon America Inc., Melville, NY) under uniform settings. Fluorescence images labeled with Fluo-4 AM were collected using an excitation wavelength of 488 nm.

### RNA isolation and qRT-PCR

Total RNA was extracted from cultured NRCMs with Trizol reagent (Invitrogen, Carlsbad, CA) according to the manufacturer's instructions. Rat-specific primers for NPPA, NPPB, β-MHC, ERO1α and GAPDH (listed in **Supplementary Table S1**) were synthesized by Invitrogen. 1 μg of total RNA was reverse transcribed into first-strand cDNA using a one-step RT kit (Takara Biotechnology, Dalian, CN). The mRNA expression levels were determined using a SYBR-Green Quantitative PCR Kit (Takara Biotechnology, Dalian, CN) on an ABI StepOne™ real-time PCR system (Thermo Fisher Scientific Inc., Carlsbad, CA). All PCR reactions were done in duplicate. GAPDH served as an endogenous control. The fold change of the mRNA expression was calculated using the 2^-∆∆CT^ method.

### Statistical analysis

All data are expressed as means ± SEM. Statistical analysis was performed with GraphPad Prism 5 and Microsoft Excel 2003.Differences were analyzed by unpaired two-tailed Student's t-tests or one-way analysis of variance (ANOVA) followed by Bonferroni multiple comparison *post-hoc* tests. *p*<0.05 was considered statistically significant.

## Results

### EndoA2 is upregulated in ischemic hearts and oxygen and glucose deprived NRCMs

**Figure 1:**
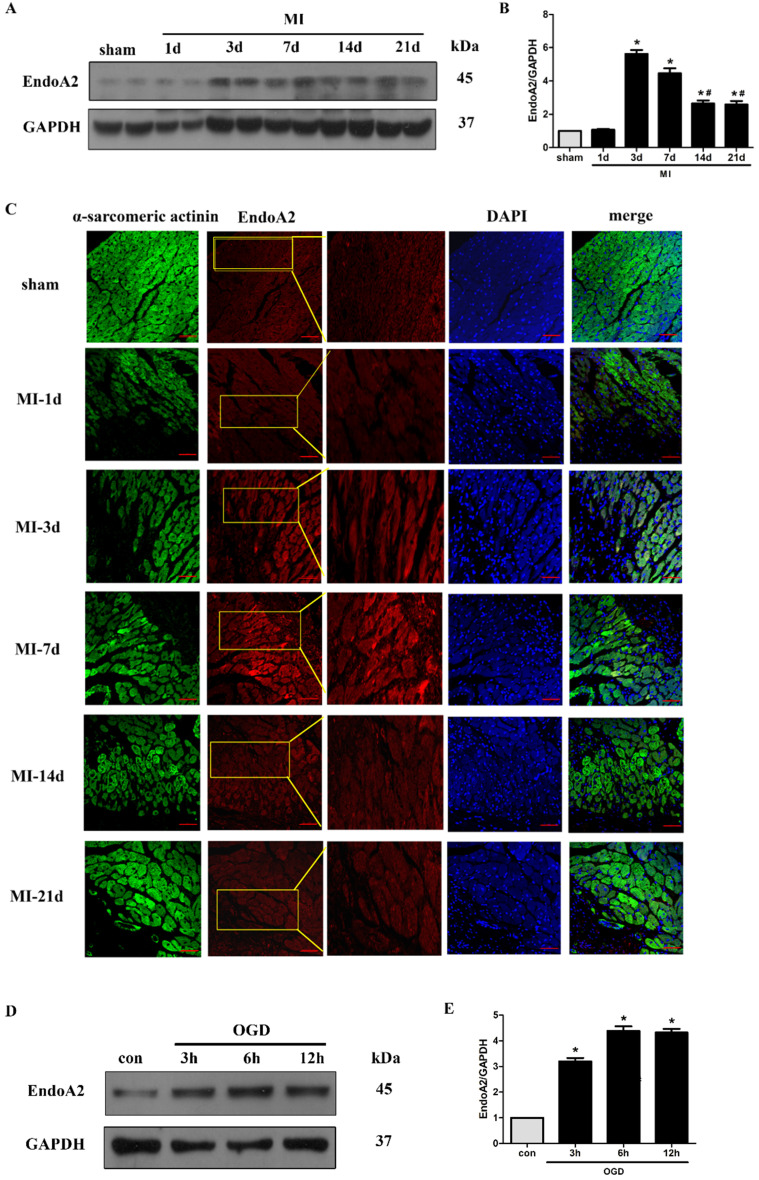
** EndoA2 is upregulated in ischemic hearts and oxygen and glucose deprived NRCMs. (A-C)** Representative western blots and images of EndoA2 expression in infarct border zone of MI mouse hearts. The protein levels of EndoA2 were acutely increased 3 days after MI. Scale bars: 50 µm (n=6, *p<0.05 vs. sham; #*p*<0.05 vs. MI-3d). **(D-E)** Western blotting results showed the expression levels of EndoA2 after OGD stimulation for different time. Densitometric analysis showed that the expression of EndoA2 was increased after OGD stimulation for 3 h (n=6, *p <0.05 vs. control).

Although we have demonstrated that EndoA2 attenuates cardiac hypertrophy in our recently published studies 19, 20, its potential in cardiac protection in response to myocardial ischemic injury has not been well characterized. Here, we established an MI mouse model by ligating the LAD coronary artery. We observed that the protein levels of EndoA2 in the infarct border zone were acutely increased 3 days after MI, and remained elevated until 21 days after MI **(Figure 1A-B)**. Notably, the protein levels of EndoA2 showed a slight decrease from 14 days compared with 3 days. Using IF staining, similar increase in EndoA2 levels was also observed in the infarct border zone **(Figure 1C)**. The increased EndoA2 was primarily localized in cardiomyocytes, which were identified by α-sarcomeric actinin staining. At the cellular level, we conducted an experiment on NRCMs with OGD stimulation which mimics ischemia. Western blotting analysis revealed that EndoA2 expression was also increased after OGD stimulation for 3 hours and remained elevated until 12 hours **(Figure 1D-E)**. These results indicate that EndoA2 is upregulated in cardiomyocytes during ischemic injury.

### EndoA2 protects the heart from MI

**Figure 2:**
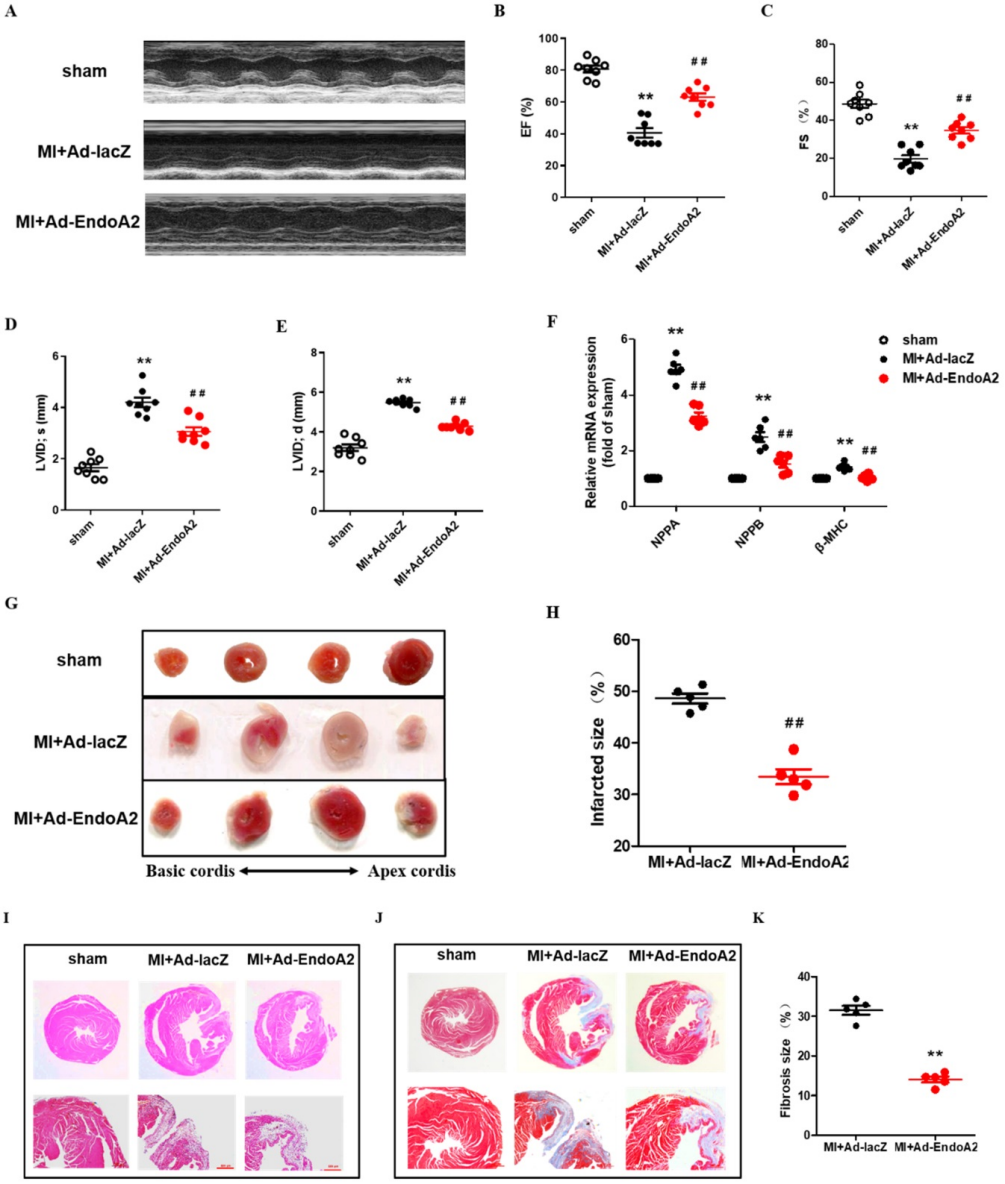
** EndoA2 protects the heart from MI. (A-E)** The representative images and analysis results of echocardiographic assessment of mouse hearts subjected to MI. Intramyocardial injection of Ad-EndoA2 abrogated the ischemia-induced decreased cardiac function. (n=8 mice, ** p <0.01 vs. sham; ##* p* <0.01 vs. MI+Ad-lacZ). **(F)** Cardiomyopathy gene expression was determined by qRT-PCR. Intramyocardial injection of Ad-EndoA2 decreased the cardiomyopathy gene expression compared with MI+Ad-lacZ group (n=8 mice, ** p <0.01 vs. sham; ##* p* <0.01 vs. MI+Ad-lacZ).** (G-H)** The representative images and analyses of TTC staining assessment of the hearts subjected to MI. Injection of Ad-EndoA2 decreased MI-induced infarct size (n=5 mice, ##* p* <0.01 vs. MI+Ad-lacZ). **(I)** The representative images of H&E staining assessment of the arrangement of striated muscle after MI. Injection of Ad-EndoA2 improved arrangement of striated muscle compared with MI+Ad-lacZ group. Scale bars: 500 µm (n=5 mice).** (J-K)** The representative images and analyses of Masson's trichrome staining assessment of fibrosis after MI. Injection of Ad-EndoA2 diminished fibrosis compared with MI+Ad-lacZ group. Scale bars: 500 µm (n=5 mice, ##* p* <0.01 vs. MI+Ad-lacZ).

To investigate the function of EndoA2 in the heart, we directly injected Ad-EndoA2 into the heart (intramyocardial injection) at the regions adjacent to the ligation site to overexpress EndoA2 under *in vivo* condition. The efficiency of Ad-EndoA2 to overexpress EndoA2 in the mouse hearts was verified by IF staining, showing significant elevation of EndoA2 in the cardiomyocytes as expected, the negative control Ad-lacZ did not affect EndoA2 level **(Supplementary Figure S1)**. We observed that both ejection fraction (EF) and fractional shortening (FS) were substantially lower in the MI+Ad-lacZ mice, compared with the sham mice **(Figure 2A-C; Supplementary Table S2)**. Moreover, left ventricular internal diameter (LVID) at end diastole and systole were both enlarged **(Figure 2D-E; Supplementary Table S2)**. Strikingly, in the MI+Ad-EndoA2 mice, the abovementioned parameters of cardiac function were all improved with elevated EF and FS, decreased LVID at end diastole and systole. We further examined the expression of molecular markers for cardiomyopathy, including natriuretic peptide A (NPPA), natriuretic peptide B (NPPB) and β-myosin heavy chain (β-MHC) **(Figure 2F)**. MI-induced expression of the genes mentioned above was reduced by Ad-EndoA2 injection. These results demonstrate that EndoA2 exerts protective effects on the cardiac function of MI mice.

Then, we preformed histological analysis. The MI mice that received the injection of Ad-EndoA2 into their hearts exhibited smaller infarct size than that of Ad-lacZ injection **(Figure 2G)**. Quantification of infarct size confirmed this observation **(Figure 2H)**. H&E staining showed disordered striated muscle and enlarged heart of MI+Ad-lacZ hearts **(Figure 2I)**. Adenovirus carrying EndoA2 gene transfer resulted in the improved arrangement of the striated muscle and heart size. As shown in **Figure 2J-K**, MI+Ad-lacZ hearts showed obvious fibrosis, and a diminished fibrosis area was observed in Ad-EndoA2-injected MI hearts. Taken together, the data presented above suggest that EndoA2 reduces MI-induced cardiac injury and preserves cardiac function.

### EndoA2 attenuates ischemia-induced cardiomyocyte apoptosis in MI mice

**Figure 3:**
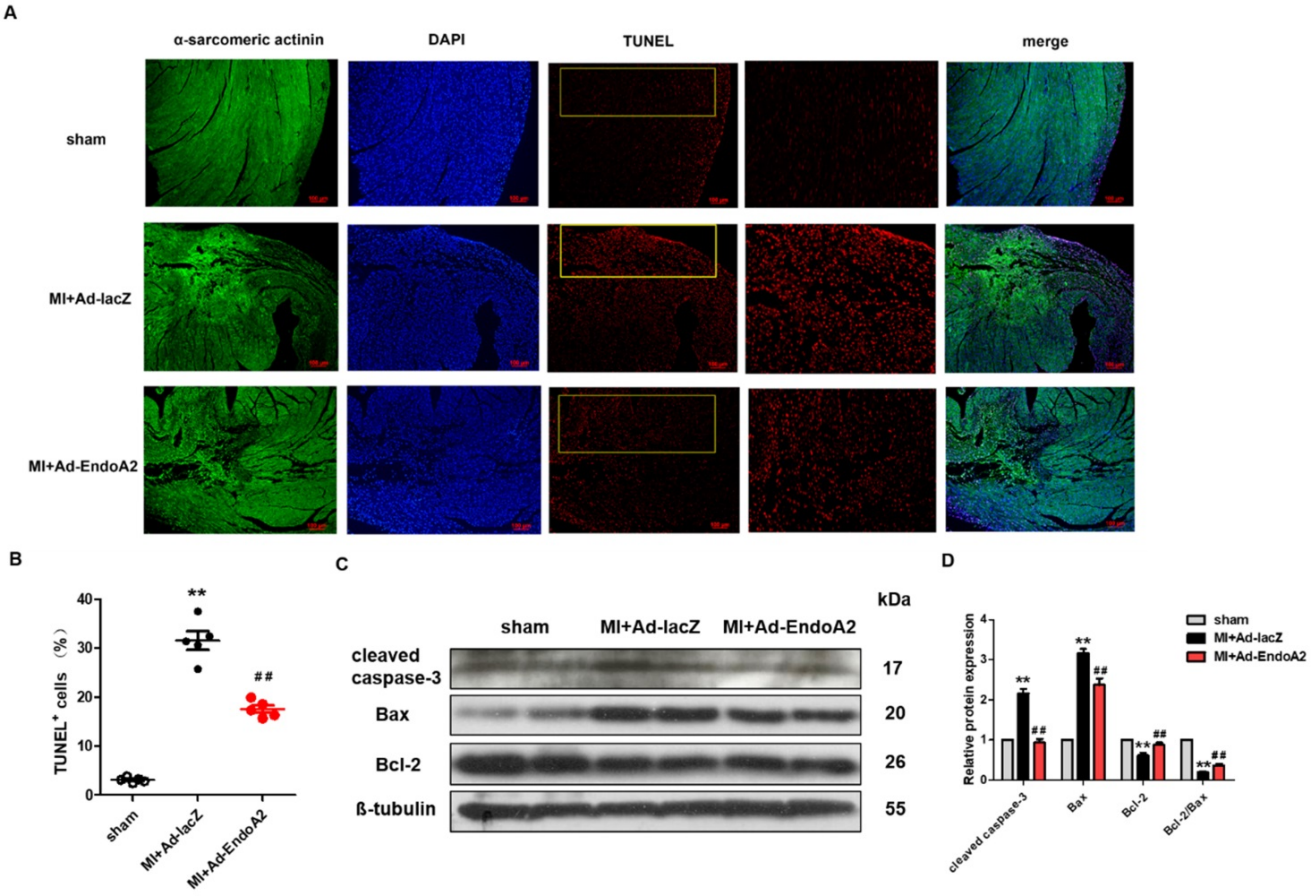
** EndoA2 attenuates ischemia-induced cardiomyocyte apoptosis in MI mice. (A)** TUNEL staining (red) on transverse sections of mouse hearts injected with Ad-EndoA2 or Ad-lacZ after MI. Scale bars: 100 µm (n=5 mice). **(B)** Quantification of TUNEL-positive cardiomyocytes. Injection of Ad-EndoA2 significantly decreased cardiomyocyte apoptosis induced by LAD ligation (n=5 mice, ** p <0.01 vs. sham; ##* p* <0.01 vs. MI+Ad-lacZ). **(C-D)** Western blotting results showed that Ad-EndoA2 inhibited cardiomyocyte apoptosis in the infarct border zone of ischemic hearts, which decreased the expression of cleaved caspase-3, Bax and increased the ratio of Bcl-2/Bax and Bcl-2 expression compared with MI+Ad-lacZ group (n=5, ** p <0.01 vs. sham; ##* p* <0.01 vs. MI+Ad-lacZ).

Numerous studies have reported that MI-induced apoptosis is detrimental to heart repair 26. To explore the role of EndoA2 in cardiomyocyte apoptosis after MI, we performed TUNEL staining and western blotting to assess apoptosis. Using TUNEL assay, we assessed apoptosis in infarcted hearts injected with either Ad-EndoA2 or Ad-lacZ. We found a substantial decrease in TUNEL signals in Ad-EndoA2 injected hearts **(Figure 3A)**. Quantification verified that apoptosis was decreased in cardiomyocytes **(Figure 3B)**. This observation was further supported by the western blotting results. As anticipated, **Figure 3C-D** showed that in the infarct border zone of MI mouse hearts, adenovirus carrying EndoA2 gene transfer resulted in decreased apoptosis-related genes, as evidenced by the reduced expression of cleaved caspase-3 and Bax and the elevated ratio of Bcl-2/Bax and Bcl-2 expression, compared with those of Ad-lacZ injected hearts. Together, these results demonstrate that the reduction of cardiomyocyte apoptosis plays a crucial role in EndoA2-mediated cardiac protection in response to MI.

### EndoA2 attenuates OGD-induced NRCM apoptosis

**Figure 4:**
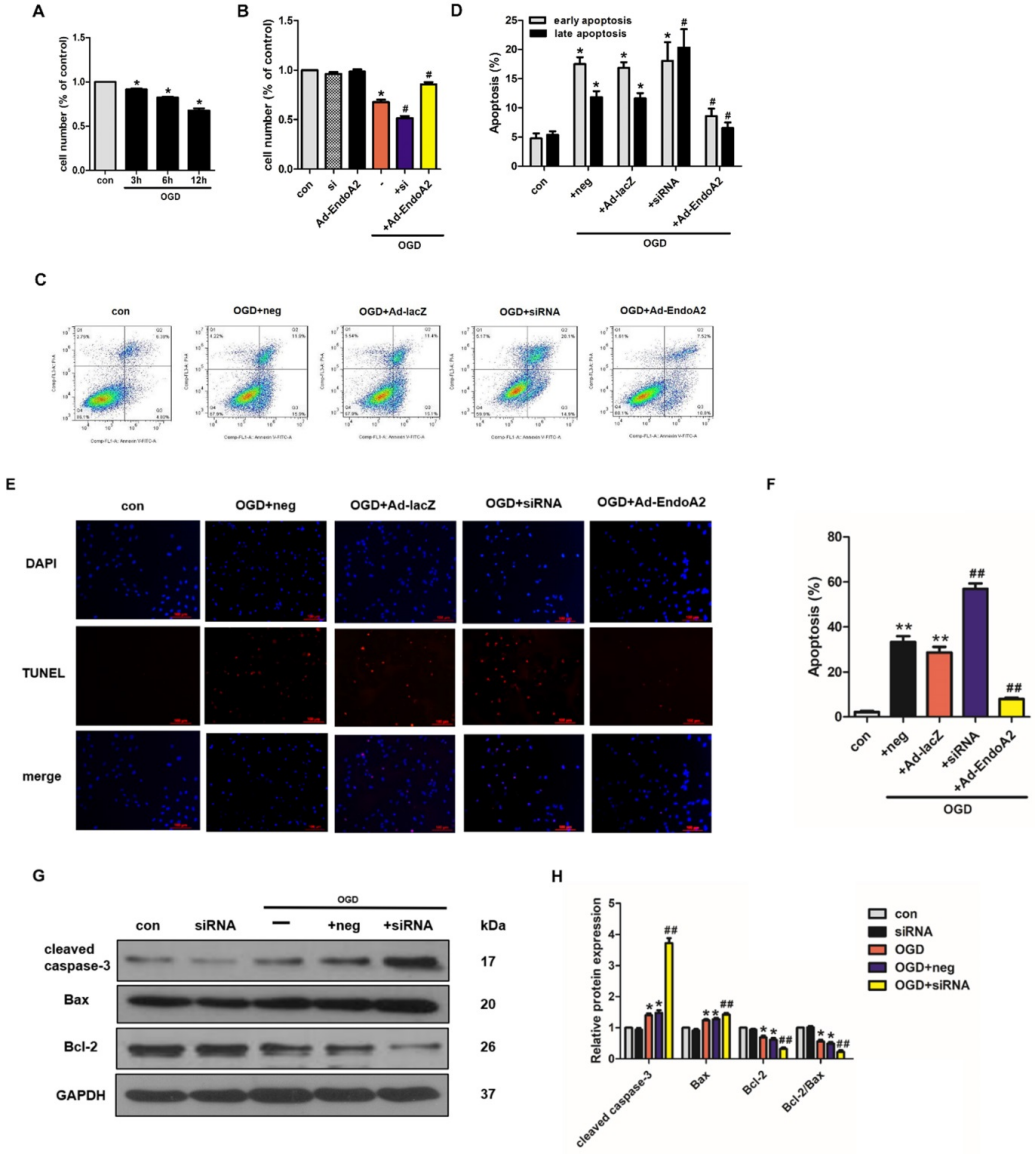

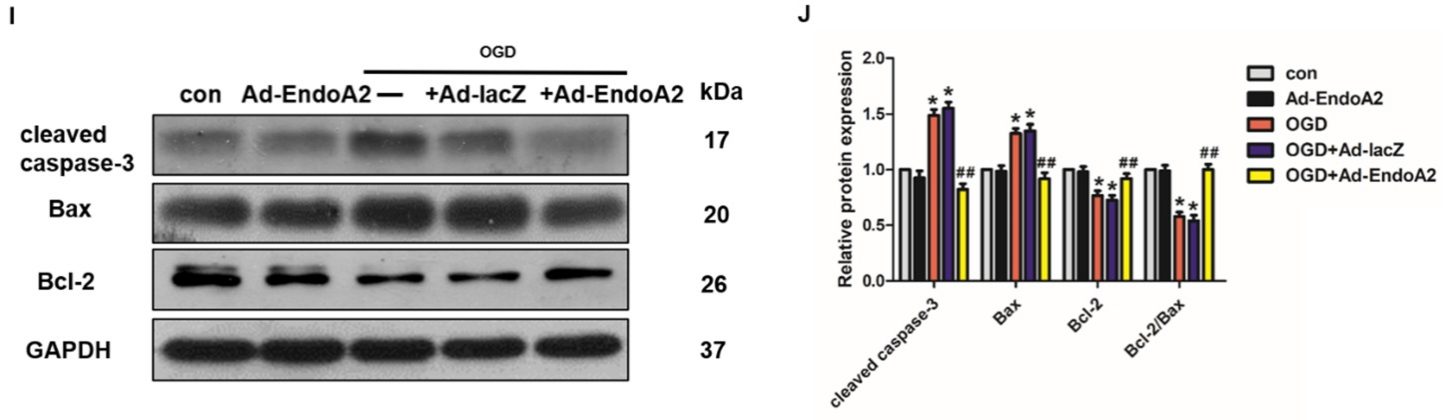
** EndoA2 attenuates OGD-induced NRCM apoptosis. (A)** CCK-8 results showed that cell number was time-dependently decreased after OGD stimulation (n=6, *p <0.05 vs. control). **(B)** CCK-8 results showed that cell number was obviously decreased after OGD stimulation for 12 h. EndoA2 overexpression increased cell number, whereas EndoA2 siRNA knockdown further decreased cell number compared with OGD stimulation (n=6, *p <0.05 vs. control, #*p* <0.05 vs. OGD). **(C)** Annexin V-FITC/PI flow cytometry analyses of OGD-induced apoptosis in cells with transfection of Ad-EndoA2 or EndoA2 siRNA. In each plot, viable cells are in the lower left quadrant, early apoptotic cells are in the lower right quadrant, and the upper right represents necrotic or late apoptotic cells. **(D)** Percentage of apoptotic cells was determined by quantitative analyses. EndoA2 overexpression decreased, whereas EndoA2 siRNA knockdown enhanced the apoptotic rate induced by OGD. Ad-lacZ and negative control (neg) had no significant effect on OGD-induced cell apoptosis (n=6, *p <0.05 vs. control, #*p* <0.05 vs. OGD+neg or OGD+Ad-lacZ). **(E)** Representative images of TUNEL and DAPI double-staining in cardiomyocytes with different treatments. The first line images represented the DAPI as nuclei and in blue color; the second line images represented the TUNEL as apoptotic nuclei and in red color; the third line images were the combination of the blue and red images. Scale bars: 100 µm.** (F)** The number of apoptotic nuclei was counted and densitometric analyses showed that OGD stimulation increased the number of apoptotic nuclei, which was decreased by EndoA2 overexpression, but further increased by EndoA2 siRNA knockdown (n=6, **p <0.01 vs. control, ##*p* <0.01 vs. OGD+neg or OGD+Ad-lacZ). **(G-J)** The effects of EndoA2 overexpression or siRNA knockdown on OGD-induced Bax, Bcl-2 and cleaved caspase-3 expression. Densitometric analyses showed that OGD stimulation decreased the expression of Bcl-2 and the ratio of Bcl-2/Bax, whereas increased the expression of Bax and cleaved caspase-3. Overexpression of EndoA2 abrogated the effects of OGD on these proteins, whereas silence of EndoA2 enhanced them (n=6, *p <0.05 vs. control, ##*p* <0.01 vs. OGD).

The effects of EndoA2 on ischemia-induced cardiomyocyte apoptosis described above indicate that EndoA2 exerts a protective effect against cardiomyocyte apoptosis. To further test this notion, NRCMs were transfected with EndoA2 siRNA **(Supplementary Figure S2A)** or Ad-EndoA2 **(Supplementary Figure S2B)** to knockdown or overexpress EndoA2 expression respectively, and then were stimulated with OGD. Cell number was measured by the CCK-8 assay. As shown in **Figure 4A**, the cell number decreased in a time-dependent manner after OGD stimulation. OGD stimulation for 12 hours caused the cell number to decrease to 67.8±5.3% compared with control. Based on our previous results that the protein expression of EndoA2 obviously increased after OGD stimulation for 12 hours **(Figure 1D-E)**, we chose OGD stimulation for 12 hours for the following experiments. To explore the role of EndoA2 on OGD-induced cell apoptosis, the cell number was subsequently determined. As **Figure 4B** showed, compared with OGD stimulation, EndoA2 siRNA knockdown further decreased the cell number from 67.8±5.3% to 51.6±4.8%, whereas EndoA2 overexpression alleviated OGD-induced cell apoptosis and increased the cell number to 85.8±4.9%.

To confirm the protective role of EndoA2 on cardiomyocyte apoptosis, additional experiments were conducted, including flow cytometry analysis of Annexin V-FITC/PI double staining, TUNEL staining and western blotting. The flow cytometry results after Annexin V-FITC/PI double staining showed that OGD stimulation increased cell apoptosis to 29.32±1.11% compared with control **(Figure 4C-D, Supplementary Figure S2C-D)**. SiRNA-mediated knockdown of EndoA2 further increased OGD-induced cell apoptosis to 38.38±3.17%, whereas EndoA2 overexpression decreased OGD-induced cell apoptosis to 15.11±1.11%. Moreover, TUNEL staining results also showed that siRNA-mediated knockdown of EndoA2 enhanced OGD-induced cell apoptosis** (Figure 4E-F, Supplementary Figure S2E-F)**. Contrary to the effects of EndoA2 knockdown, EndoA2 overexpression alleviated cell apoptosis induced by OGD stimulation.

Subsequently, the expression of apoptosis-related protein expression was monitored by measuring Bax, cleaved caspase-3 and Bcl-2 levels. As **Figure 4G-J** showed, OGD stimulation obviously increased the expression of Bax and cleaved caspase-3 and decreased the expression of Bcl-2 and the ratio of Bcl-2/Bax compared with control. SiRNA-mediated knockdown of EndoA2 further induced an increase in Bax and cleaved caspase-3 expression, but further induced a decrease in Bcl-2 expression and the Bcl-2/Bax ratio, compared with OGD stimulation. EndoA2 overexpression led to the opposite effects and reversed the changes in protein expression caused by OGD stimulation. These evidences together demonstrate that EndoA2 inhibits OGD-induced cardiomyocyte apoptosis.

### EndoA2 suppresses ER stress

**Figure 5:**
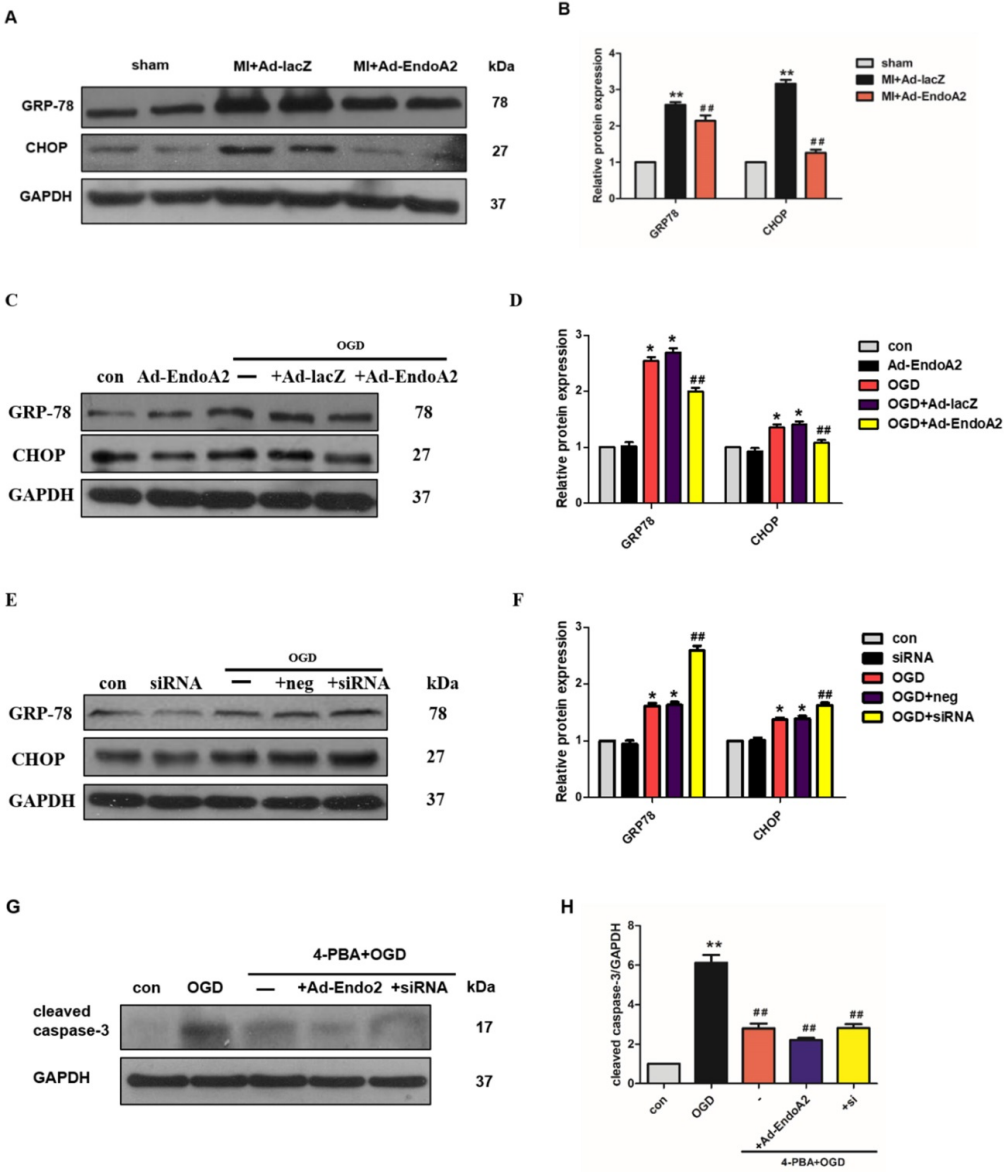
** EndoA2 suppresses ER stress. (A-B)** Western blotting results showed that Ad-EndoA2 inhibited ER stress in the border of ischemic hearts (n=5, ** p <0.01 vs. sham; ##* p* <0.01 vs. MI+Ad-lacZ). **(C-D)** Western blotting results showed the expression of GRP-78 and CHOP after transfection of Ad-EndoA2. Densitometric analyses showed that EndoA2 overexpression decreased the expression of GRP-78 and CHOP induced by OGD (n=6, * p <0.05 vs. control, ##* p* <0.01 vs. OGD). **(E-F)** Western blotting results showed the expression of GRP-78 and CHOP after transfection of EndoA2 siRNA. Densitometric analyses showed that EndoA2 siRNA knockdown further increased the expression of GRP-78 and CHOP induced by OGD (n=6, * p <0.05 vs. control, ##* p* <0.01 vs. OGD). **(G-H)** Western blotting results showed the expression of cleaved caspase-3 after pretreatment with 4-PBA in NRCMs. Densitometric analyses showed that the increased levels of cleaved caspase-3 induced by OGD were reversed by pretreatment with 4-PBA (n=6, ** p <0.01 vs. control, ##* p* <0.01 vs. OGD).

It has been reported that suppression of ER stress is beneficial for attenuating cardiomyocyte apoptosis 27. ER stress induces the apoptotic pathway by stimulating the transcriptional activation of the pro-apoptotic transcriptional factor C/EBP homologous protein (CHOP). We therefore proceeded to test whether ER stress played a critical role in EndoA2-mediated attenuation of cardiomyocyte apoptosis in response to MI. We investigated the protein expression of ER stress markers, including GRP-78 and CHOP. In MI mice, Ad-EndoA2-injected hearts exhibited significantly reduced ER stress responses in the infarct border zone, as revealed by the decreased expression of GRP-78 and CHOP **(Figure 5A-B)**, compared with MI+Ad-lacZ-injected hearts. These data reveal that attenuated ER- stress-induced apoptosis is at least partially responsible for the cardiac protection of EndoA2 in response to MI.

Next, we detected the protein levels of GRP-78 and CHOP in NRCMs. As expected, the protein levels of GRP-78 and CHOP were significantly increased in the NRCMs after OGD stimulation **(Figure 5C-F)**, indicating that the ER stress response was acutely activated. EndoA2 overexpression suppressed the ER stress response, as indicated by the remarkably decreased GRP-78 and CHOP expression. In contrast, siRNA-mediated EndoA2 knockdown further increased OGD-induced expression of GRP-78 and CHOP. To further assess the involvement of ER stress in the EndoA2-mediated suppression of cardiomyocyte apoptosis, NRCMs were treated with the ER stress inhibitor 4-PBA. Western blotting results showed that the increased level of cleaved caspase-3 induced by OGD and EndoA2 knockdown was reversed by pretreatment with 4-PBA **(Figure 5G-H)**. These data suggests that the suppression of ER stress is responsible for the anti-apoptotic effect of EndoA2 in cardiomyocytes after ischemic injury.

### Inactivation of the Ca^2+^/calcineurin/NFAT signaling pathway is essential for the cardioprotective effects of EndoA2

**Figure 6:**
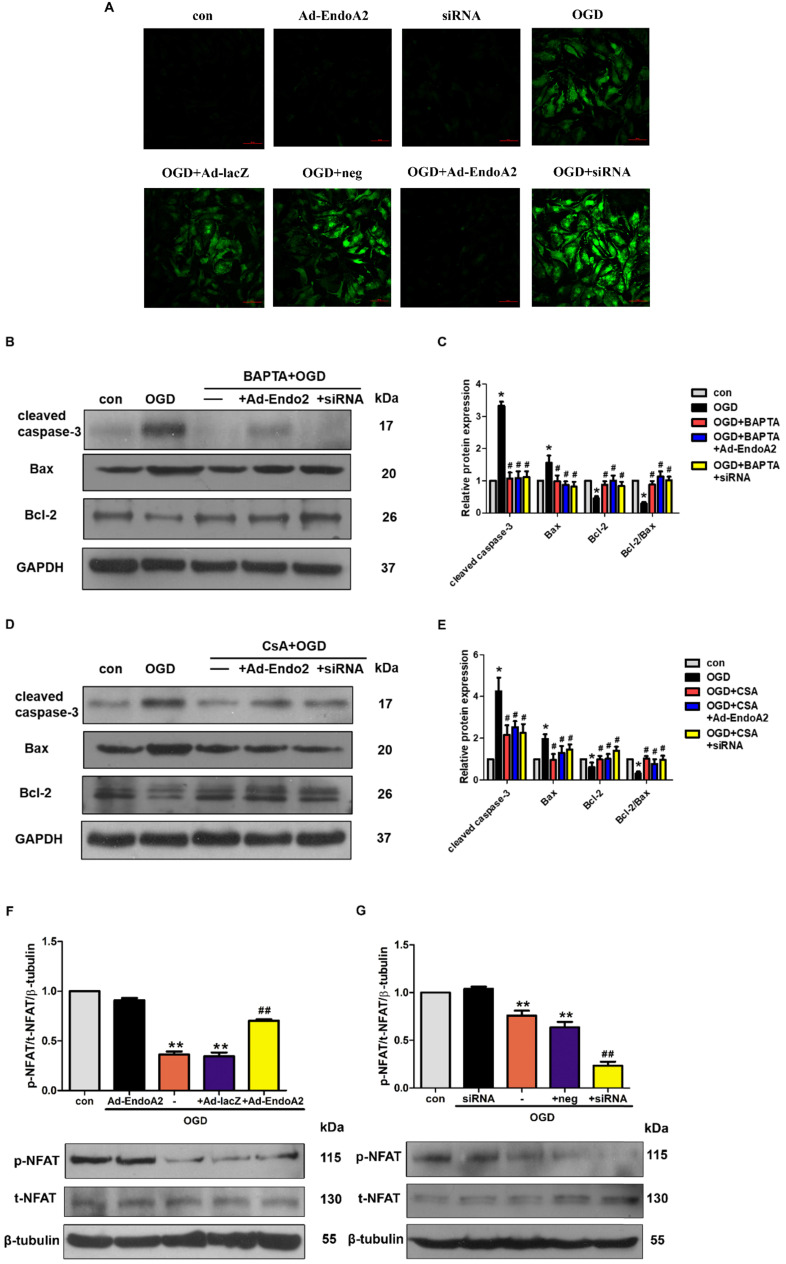
** Inactivation of Ca^2+^/calcineurin/NFAT signaling pathway is essential for the cardioprotective effects of EndoA2. (A)** NRCMs were transfected with Ad-EndoA2 or EndoA2 siRNA in the absence or presence of OGD stimulation for 12h, then fluo-4 AM calcium staining was performed. The images were detected by fluorescent microscope. The representative images showed that intracellular Ca^2+^ level obviously increased after OGD stimulation, which was further enhanced by EndoA2 siRNA knockdown, while attenuated by EndoA2 overexpression. Scale bars: 50 µm (n=6). **(B-C)** Western blotting results showed the expression of cleaved caspase-3, Bax and Bcl-2 after being treated with BAPTA. Densitometric analyses showed that the change of apoptosis-related protein induced by OGD stimulation was reversed by pretreatment with BAPTA (n=6, *p <0.05 vs. control, #*p* <0.05 vs. OGD). **(D-E)** Western blotting results showed the expression of cleaved caspase-3, Bax and Bcl-2 after being treated with CSA. Densitometric analyses showed that the change of apoptosis-related protein by OGD was reversed by pretreatment with CSA (n=6, *p <0.05 vs. control, #*p* <0.05 vs. OGD). **(F-G)** EndoA2 attenuated the decrease of p-NFAT induced by OGD, while EndoA2 siRNA knockdown further strengthened the decrease of p-NFAT induced by OGD. Total NFAT was not changed under the indicated conditions (n=6, **p <0.01 vs. control, ##*p* <0.01 vs. OGD).

ER controls many cellular responses and signaling transduction pathways in response to stress through the transport of Ca^2+^ in and out of the ER lumen. The disruption of ER calcium homeostasis induces cell apoptosis 28. To elucidate the molecular mechanism underlying EndoA2-mediated cardiac protection, we investigated Ca^2+^/calcineurin/NFAT pathway. Previous studies indicate that intracellular Ca^2+^ level is increased in the ischemic myocardium 29, 30, thus, we examined whether Ca^2+^ was involved in the cardiac protection of EndoA2. In agreement with previous studies, OGD stimulation obviously increased the intracellular Ca^2+^ level **(Figure 6A)**. Compared with OGD treatment stimulation, knockdown of EndoA2 further increased the intracellular Ca^2+^ level. Conversely, EndoA2 overexpression decreased the intracellular Ca^2+^ level. To further determine the relationship between the Ca^2+^/calcineurin signaling pathway and EndoA2-mediated cardiac protection after myocardial ischemic injury, the Ca^2+^ chelator BAPTA and the calcineurin inhibitor CsA were used. Pretreatment of NRCMs with BAPTA remarkably reduced OGD-induced cleaved caspase-3 expression, and abrogated the effect of EndoA2 knockdown **(Figure 6B-C)**. Pretreatment with CsA created similar reduction of cleaved caspase-3 expression as BAPTA **(Figure 6D-E)**.

Activated calcineurin directly binds to NFAT transcription factors, resulting in NFAT dephosphorylation and nuclear translocation^31^. Thus, we measured the phosphorylation level of NFAT. NFAT was significantly dephosphorylated by OGD stimulation, and this effect was further enhanced by siRNA-mediated EndoA2 knockdown but reversed by EndoA2 overexpression **(Figure 6F-G).** The results collectively indicate that the suppression of the Ca^2+^/calneurin/NFAT signaling pathway contributes to the protective effects of EndoA2 in myocardial ischemic injury.

### ERO1α-mediated stimulation of IP_3_R activity is critical for ER Ca^2+^ release

**Figure 7:**
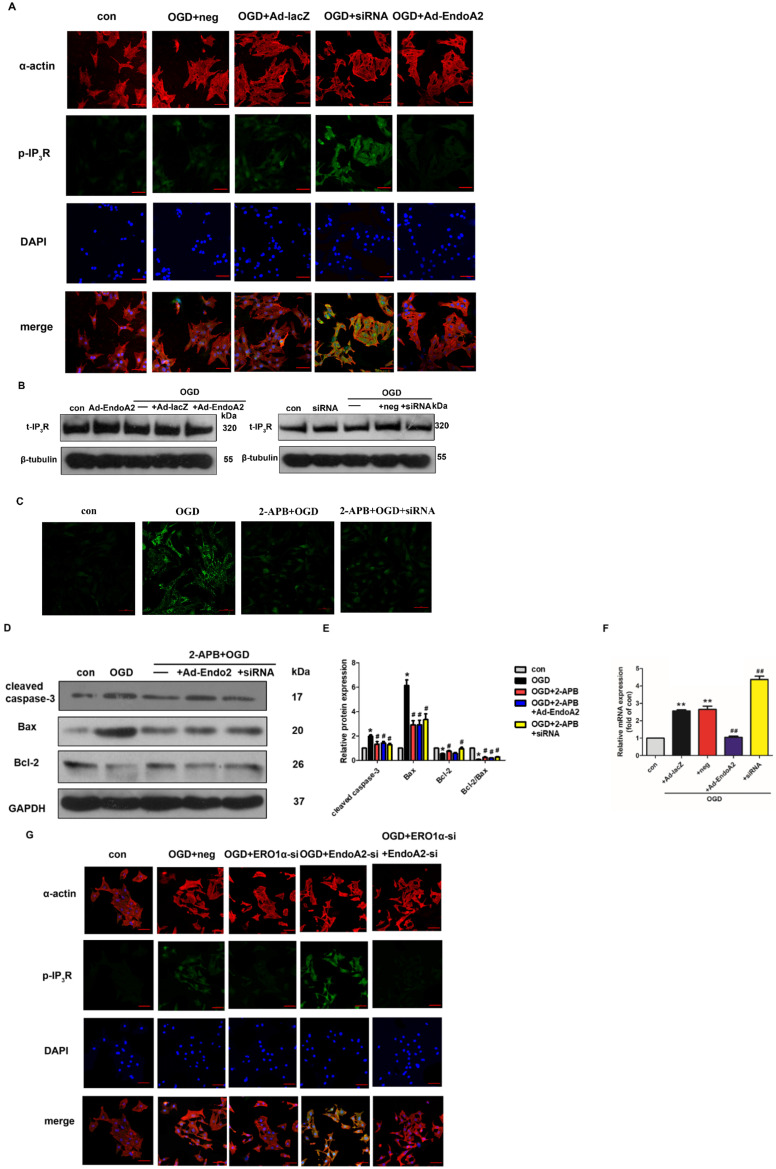

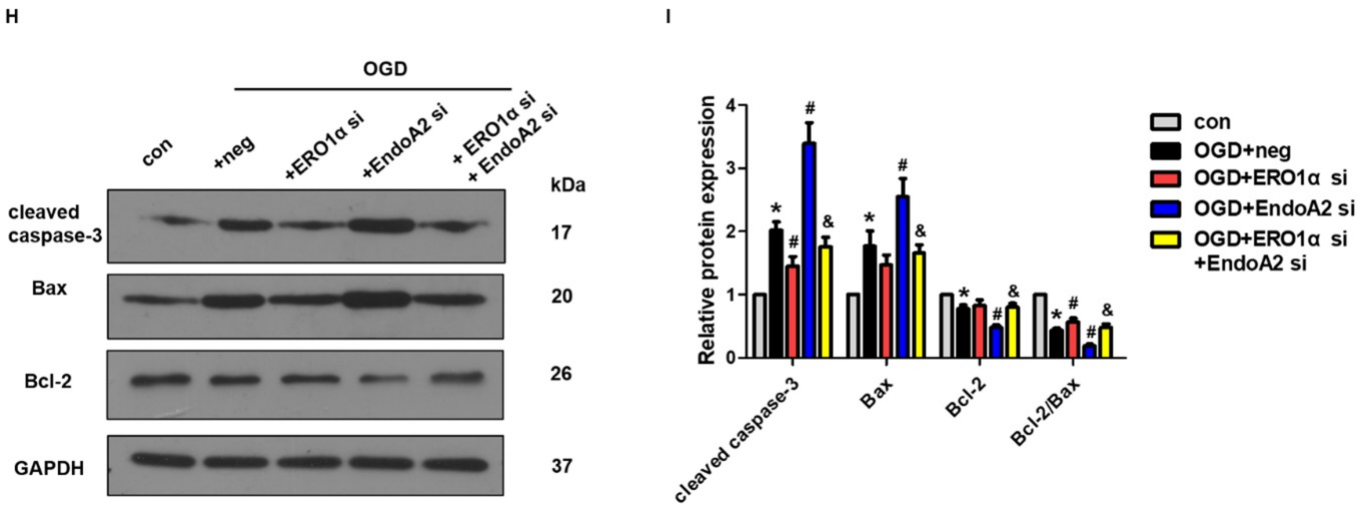
** ERO1α-mediated stimulation of IP_3_R activity is critical for ER Ca^2+^ release**. **(A)** Representative images of p-IP3R showed that OGD treatment increased the phosphorylation of IP_3_R, which was reversed by EndoA2 overexpression and further strengthened by EndoA2 siRNA knockdown. Scale bars: 50 µm (n=6).** (B)** Representative images of t-IP_3_R showed that t-IP_3_R was not changed under the indicated conditions (n=4). **(C)** Pretreatment with 2-APB inhibited intracellular Ca^2+^ release induced by OGD treatment. Scale bars: 50 µm. (n=6). **(D-E)** Western blotting results showed the expression of cleaved caspase-3, Bax and Bcl-2 after being treated with 2-APB. Densitometric analyses showed that the change of apoptosis-related protein induced by OGD was reversed by pretreatment with 2-APB (n=6, * p <0.05 vs. control, #* p* <0.05 vs. OGD). **(F)** qRT-PCR detection of ERO1α mRNA expression. EndoA2 overexpression decreased ERO1α mRNA expression induced by OGD, while EndoA2 siRNA knockdown further increased it (n=6, ** p <0.01 vs. control, ##* p* <0.01 vs. OGD+Ad-lacZ or OGD+neg). **(G)** Representative images showed that ERO1α siRNA knockdown attenuated the phosphorylation of IP_3_R induced by OGD or EndoA2 siRNA knockdown. Scale bars: 50 µm (n=6). **(H-I)** Western blotting results showed that ERO1α siRNA knockdown reversed the change of apoptosis-related protein induced by OGD or EndoA2 siRNA knockdown (n=6, * p <0.05 vs. control, #* p* <0.05 vs. OGD+neg, &* p* <0.05 vs. OGD+EndoA2 si).

Based on a previous study showing that CHOP targets ERO1α to activate ER Ca^2+^ release though IP_3_R 25, we hypothesized that EndoA2 attenuated myocardial ischemic injury by suppressing the ERO1α-mediated stimulation of IP_3_R activity. IP_3_R, which is located in the ER, regulates Ca^2+^ release from the ER. To determine the role of IP_3_R in myocardial ischemic injury, we examined the activity of IP_3_R. As shown in **Figure 7A-B and Supplementary Figure S3**, OGD stimulation increased the phosphorylation level of IP_3_R without changing its total expression level, this effect was reversed by EndoA2 overexpression but further enhanced by siRNA-mediated EndoA2 knockdown. Pretreatment with the IP_3_R inhibitor 2-APB obviously attenuated the intracellular Ca^2+^ release and cardiomyocyte apoptosis induced by OGD stimulation and EndoA2 knockdown** (Figure 7C-E)**. To better understand the molecular mechanism, we performed qRT-PCR to examine ERO1α mRNA expression. The increased ERO1α gene expression induced by OGD stimulation was diminished by EndoA2 overexpression but augmented by siRNA-mediated EndoA2 knockdown **(Figure 7F).** Then siRNA was used to decrease the ERO1α protein level **(Supplementary Figure S4)**. We found that knockdown of ERO1α caused a significant reduction in OGD-induced phosphorylation level of IP_3_R and abrogated the effect of EndoA2 knockdown **(Figure 7G, Supplementary Figure S5)**. Furthermore, knockdown of ERO1α attenuated OGD-induced cell apoptosis-related protein, and reversed the effect of EndoA2 knockdown **(Figure 7H-I)**. These data indicates that ERO1α plays an important role in ER-stress-induced, CHOP-dependent apoptosis in cardiomyocytes. The cardioprotective effect of EndoA2 is dependent on ERO1α and acts upstream of ERO1α.

## Discussion

**Figure 8:**
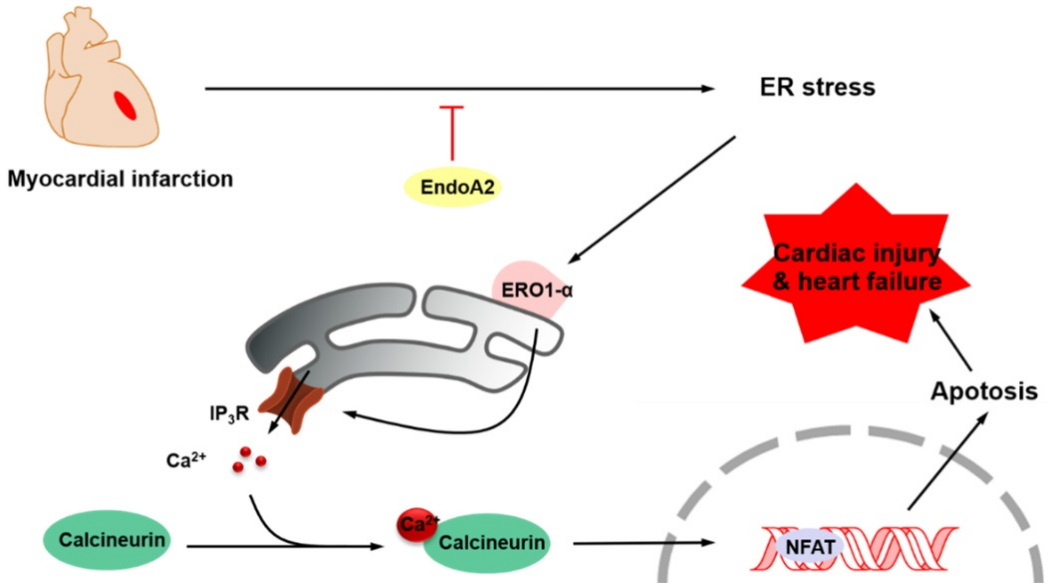
** Schematic model of ER stress/ERO1α/IP_3_R signaling pathway mediated by EndoA2.** EndoA2 attenuates ER stress, which in turn leads to downregulation of GRP78 and CHOP, followed by inactivation of ERO1α/IP_3_R signaling pathway. Thus, EndoA2 ameliorates cardiomyocyte apoptosis and preserves cardiac function upon ischemic injury.

The aim of this study was to elucidate the role of EndoA2 and to delineate the underlying mechanism in myocardial ischemic injury. Our data demonstrated that intramyocardial injection of Ad-EndoA2 into mice with MI preserved cardiac function during ischemic injury. Moreover, overexpression of EndoA2 mitigated postinfarction-induced cardiomyocyte apoptosis. Furthermore, at the cellular level, EndoA2 overexpression attenuated OGD-induced cardiomyocyte apoptosis by suppressing ER stress, while EndoA2 knockdown further promoted OGD-induced cardiomyocyte apoptosis. At the subcellular level, EndoA2 knockdown deleteriously increased the intracellular Ca^2+^ levels in NRCMs to increase apoptosis, possibly through the Ca^2+^/calcineurin/NFAT pathway. Finally, evidence was obtained to support the notion that ERO1α-mediated stimulation of IP_3_R activity accounted for the increased intracellular Ca^2+^ level in NRCMs in which endogenous EndoA2 was knocked down. These findings allowed us to propose the following paradigm for the regulation of cardiac protection of EndoA2: EndoA2 →↓ ER stress →↓ ERO1α/IP_3_R →↓ ER Ca^2+^ release →↓ cardiomyocyte apoptosis →↓myocardial ischemic injury **(Figure 8)**. Based on these findings, we conclude that EndoA2 protects the heart in response to MI by suppressing the ERO1α-mediated stimulation of IP_3_R activity.

EndoA2, which was originally identified as a fusion partner of mixed-lineage leukemia gene in childhood leukemia 32, is involved in vesicle endocytosis. Our previous study verified that EndoA2 promoted H_2_O_2_-induced rat basilar artery smooth muscle cell apoptosis 18, Moreover, EndoA2 inhibited H_2_O_2_-induced H9C2 cardiomyocyte apoptosis by enhancing autophagy 21 and attenuated cardiac hypertrophy induced by angiotensin II 20 or isoproterenol 19. However, whether EndoA2 plays a key role in myocardial ischemic injury is not clear. In this study, we found that the expression of EndoA2 in the infarct border zone of mice with MI robustly increased 7 days after MI. Similarly, remarkably increased EndoA2 expression was observed after OGD stimulation. A question we asked was whether the upregulation of EndoA2 upon myocardial ischemic injury was detrimental or beneficial. To clarify this issue, we directly injected Ad-EndoA2 into the heart and investigated whether EndoA2 could alter cardiac function. Here, we presented evidence that EndoA2 preserved cardiac function upon ischemic injury.

Apoptosis plays a crucial role in the pathogenesis of a variety of cardiovascular diseases, including atherosclerosis 33, myocardial ischemia and reperfusion 34, diabetic cardiomyopathy 35 and MI 36. Due to a loss of terminally differentiated cardiomyocytes, which is dedicated to wall thinning, ventricular dilatation and fibrosis, apoptosis is a critical contributor to heart failure. It has been reported that TGFβR3 protected ischemia-induced cardiomyocyte apoptosis via activation p38 signaling pathway, whereas loss of TGFβR3 reduced apoptosis and improved cardiac function 37. Our recently published study verified that EndoA2 attenuated H_2_O_2_-induced rat basilar artery smooth muscle cell apoptosis 18. Moreover, EndoA2 inhibited H_2_O_2_-induced H9C2 cardiomyocyte apoptosis by enhancing autophagy 21. However, whether EndoA2 suppresses cardiomyocyte apoptosis induced by myocardial ischemia remains to be investigated. Accordingly, in this study, by using the strategies of both EndoA2 overexpression and siRNA-mediated knockdown, we identified a potential role of EndoA2 as a novel target for the treatment of myocardial ischemic injury. Overexpression of EndoA2 significantly inhibited cardiomyocyte apoptosis induced by OGD stimulation or myocardial ischemia, whereas knockdown of EndoA2 promoted the apoptosis induced by OGD stimulation, which suggested a potential target for the prevention of myocardial ischemic injury.

Apoptosis is a form of programmed cell death and includes three pathways: the death receptor pathway, the mitochondria pathway and the ER stress pathway 38. How EndoA2 attenuated cardiomyocyte apoptosis remains to be investigated. ER stress pathway has been discovered as a new apoptotic pathway in recent years. At the early and middle stages of ER stress, ER triggers an adaptive process, known as unfolded protein response (UPR), to maintain ER homeostasis. However, persistent or severe ER stress switches UPR from an adaptive response to a response that initiates apoptosis 39. During MI, GRP78 protein expression is increased near the infarct site; however, no increase is observed further away from the damage 40. Additionally, Hardy and Raiter found that cardiomyocytes that were cultured in prolonged stress condition (both hypoxia and starvation) launched an upregulation of GRP78 and an increase in cardiomyocyte apoptosis 41. Consistent with previous studies, our results demonstrated that siRNA-mediated EndoA2 knockdown significantly enhanced the OGD-induced GRP-78 expression, followed by the increased expression of CHOP. In contrast, EndoA2 overexpression led to the opposite effects. The results from MI mouse hearts were consistent with those from NRCMs. Our data established that EndoA2 acted as an anti-apoptotic protein to protect cardiomyocytes from apoptosis by attenuating ER stress.

Thus, the following question remains to be determined: how does EndoA2 regulate ER stress and cardiomyocyte apoptosis? The ER not only interferes with proper protein folding, but also functions as an important storage site for Ca^2+^. Intracellular Ca^2+^ homeostasis is crucial for cell survival, and disruption of Ca^2+^ homeostasis is involved in apoptosis 28, 42. In the pancreatic beta cells of type 1 diabetes model, streptozotocin disrupts intracellular Ca^2+^ homeostasis, leads to excessive ER stress and induces apoptosis 8. In endothelial cells, AMPKα2 deletion increases intracellular Ca^2+^ accumulation, and consequent ER stress activation and atherosclerosis 43. In cardiomyocytes, BNIP3 decreases the Ca^2+^ content in the ER by shifting Ca^2+^ from the ER to the mitochondria and contributes to myocardial systolic dysfunction 44. Our further studies found that EndoA2 overexpression attenuated the OGD-induced Ca^2+^ accumulation in cardiomyocytes, whereas siRNA-mediated EndoA2 knockdown enhanced this Ca^2+^ accumulation. Ca^2+^, which acts as a ubiquitous secondary messenger, is involved in apoptosis by regulating downstream pathway. Numerous studies have reported that the Ca^2+^/calcineurin/NFAT pathway is involved in apoptosis 45, 46. To determine if Ca^2+^/calcineurin/NFAT pathway is involved in the cardioprotective role of EndoA2, we used the Ca^2+^ chelator BAPTA and the calcineurin inhibitor CsA. The results showed that the cell apoptosis induced by EndoA2 knockdown was reversed by pretreatment with BAPTA or CsA. Moreover, the decreased level of NFAT phosphorylation induced by OGD stimulation was further enhanced by siRNA-mediated EndoA2 knockdown but reversed by EndoA2 overexpression. These observations suggested that in OGD-induced cardiomyocyte apoptosis, EndoA2 inhibited the intracellular Ca^2+^ accumulation and the downstream Ca^2+^/calcineurin/NFAT pathway to attenuate apoptosis.

An interesting finding in the present study is that EndoA2 regulates IP_3_R activity to regulate ER Ca^2+^ release. IP_3_R is a ubiquitously expressed ER Ca^2+^ channel. The Ca^2+^ release mediated by IP_3_R regulates multiple cellular processes, including apoptosis. In HeLa cells, the tumor suppressor BRCA1 directly binds to IP_3_R and increases the probability of IP_3_R opening, which is dedicated to stimulating Ca^2+^-dependent apoptosis 42. In neurons, RIP140 attenuates IP_3_R-mediated Ca^2+^ release by binding to IP_3_R to suppress its channel opening 13. Our results were consistent with previous studies. It was demonstrated that OGD increased the phosphorylation of IP_3_R, which causes increased IP_3_R activity. EndoA2 overexpression decreased IP_3_R phosphorylation, while siRNA-mediated EndoA2 knockdown further increased IP_3_R phosphorylation. Moreover, pretreatment with 2-APB, which is an inhibitor of IP_3_R, abrogated the OGD-induced intracellular Ca^2+^ accumulation and cardiomyocyte apoptosis. ERO1α, which hyperoxidizes the ER and promotes cell apoptosis, can activate IP_3_R stimulating excessive Ca^2+^ release from the ER and thereby triggering cell apoptosis. Based on previous studies showing a possible link between the redox state of the ER lumen and the activity of IP_3_R 47, 48, we tested the hypothesis that ERO1α would sensitize IP_3_R to open its channel during ER stress in MI. Most strikingly, ERO1α knockdown decreased the phosphorylation level of IP_3_R, followed by decreased apoptosis, which might account for the cardiac protection of EndoA2 in response to MI.

In conclusion, the present study identifies EndoA2 as a potential target molecule for the prevention of myocardial ischemic injury. The protective effect of EndoA2 can be explained by the inhibition of ERO1α-mediated IP_3_R activity, and the downstream Ca^2+^/calcineurin/NFAT pathway. At the heart of this pathway, the ERO1α-mediated IP_3_R pathway plays the most critical role. Apoptosis plays a crucial role in cardiac dysfunction. Our study of the attenuation of cardiomyocyte apoptosis by EndoA2 will provide a better understanding of the prevention of myocardial ischemic injury. In addition, our data provides us with new insight into the role of EndoA2 in the pathophysiological processes in heart diseases.

## Supplementary Material

Supplementary figures and table.

## Abbreviations

EndoA2: endophilin A2
IHD: ischemic heart disease
MI: myocardial infarction
NRCMs: neonatal rat cardiomyocytes
OGD: oxygen and glucose deprivation
LAD: left anterior descending coronary artery
Ad-EndoA2: adenoviral EndoA2
ER: endoplasmic reticulum
ERO1α: ER oxidoreductase 1α
IP_3_R: inositol 1, 4, 5-trisphosphate receptor
NFAT: nuclear factor of activated T-cells
CsA: Cyclosporin A
4-PBA: sodium phenylbutyrate
2-APB: 2-Aminoethoxydiphenylborate
BAPTA: 1,2-bis(o-aminophenoxy)ethane-N,N,N′,N′-tetraacetic acid
TUNEL: terminal deoxynucleotidyl transferase dUTP nick-end labeling
IF: Immunofluorescence
EF: ejection fraction
FS: fractional shortening
LVID: left ventricular internal diameter
CHOP: C/EBP homologous protein
