# Supplementary material for: Endophilin A2-mediated alleviation of endoplasmic reticulum stress-induced cardiac injury involves the suppression of ERO1α/IP3R signaling pathway
Source: Int J Biol Sci. 2021 Aug 26;17(13):3672–88. doi: 10.7150/ijbs.60110 (PMC8416715; doi:10.7150/ijbs.60110)
Supplement: Supplementary file 1 — Supplementary figures and table. [file ijbsv17p3672s1.pdf]

Supplementary Table

Gene	Forward sequence	Reverse sequence
NPPA	GGGCTTCTTCCTCTTCCTG	CGCTTCATCGGTCTGCTC
NPPB	GAACAATCCACGATGCAGAA	CCTTGGTCCTTTGAGAGCTG
β-MHC	GAGACGGACGCCATACAG	CCACATCCACCATCAGGT
ERO1α	CTGCGTCGGCTGCTTCAA	TAACTGGGTCCGCTTTCC
GAPDH	GCAAGTTCAACGGCACAG	GCCAGTAGACTCCACGACAT

Table S1. Rat-specific primer sequences for qPCR.

	sham (n=8)	MI+Ad-lacZ (n=8)	MI+Ad-EndoA2 (n=8)
LVAW;s (mm)	1.39±0.04	0.77±0.16**	1.46±0.1##
LVAW;d (mm)	0.77±0.06	0.53±0.07**	0.71±0.09
LVPW;s (mm)	1.41±0.08	1.14±0.12	1.23±0.12
LVPW;d (mm)	0.85±0.07	0.73±0.08	0.82±0.08
CO (%)	27.24±2.08	13.25±3.62**	27.86±2.51##
LVID;s (mm)	1.65±0.13	4.20±0.18**	3.06±0.19##
LVID;d (mm)	3.19±0.16	5.47±0.10**	4.30±0.11##
EF (%)	80.83±2.12	38.10±4.45**	63.16±2.69##
FS (%)	48.68±2.14	18.86±2.45**	34.37±1.93##

Table S2. Echocardiography analyses of cardiac function after intramyocardial injection of Ad-EndoA2 or Ad-lacZ post-MI. LVAW;s: left ventricular anterior wall at end systolic; LVAW;d: left ventricular anterior wall at end diastolic; LVPW;s: left ventricular posterior wall at end systolic; LVPW;d: left ventricular posterior wall at end diastolic; CO: cardiac output; LVID;s: left ventricular internal diameter at end systolic; LVID;d: left ventricular internal diameter at end diastolic; EF: ejection fraction; FS: fractional shortening (n=8 mice, ***p*<0.01 vs. sham group, ##*p*<0.01 vs. MI+Ad-lacZ group).

Supplementary Figure

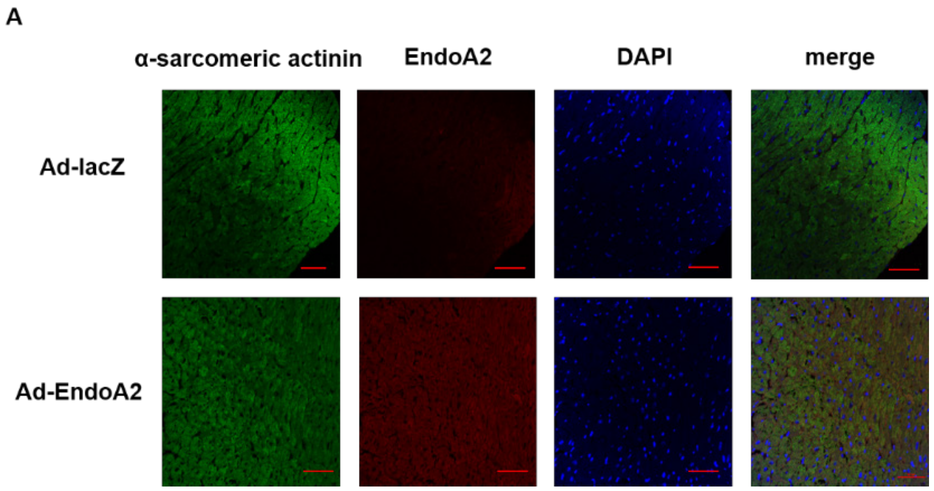

Figure S1. The gene overexpression effects of Ad-EndoA2 after intramyocardial injection (n=3).

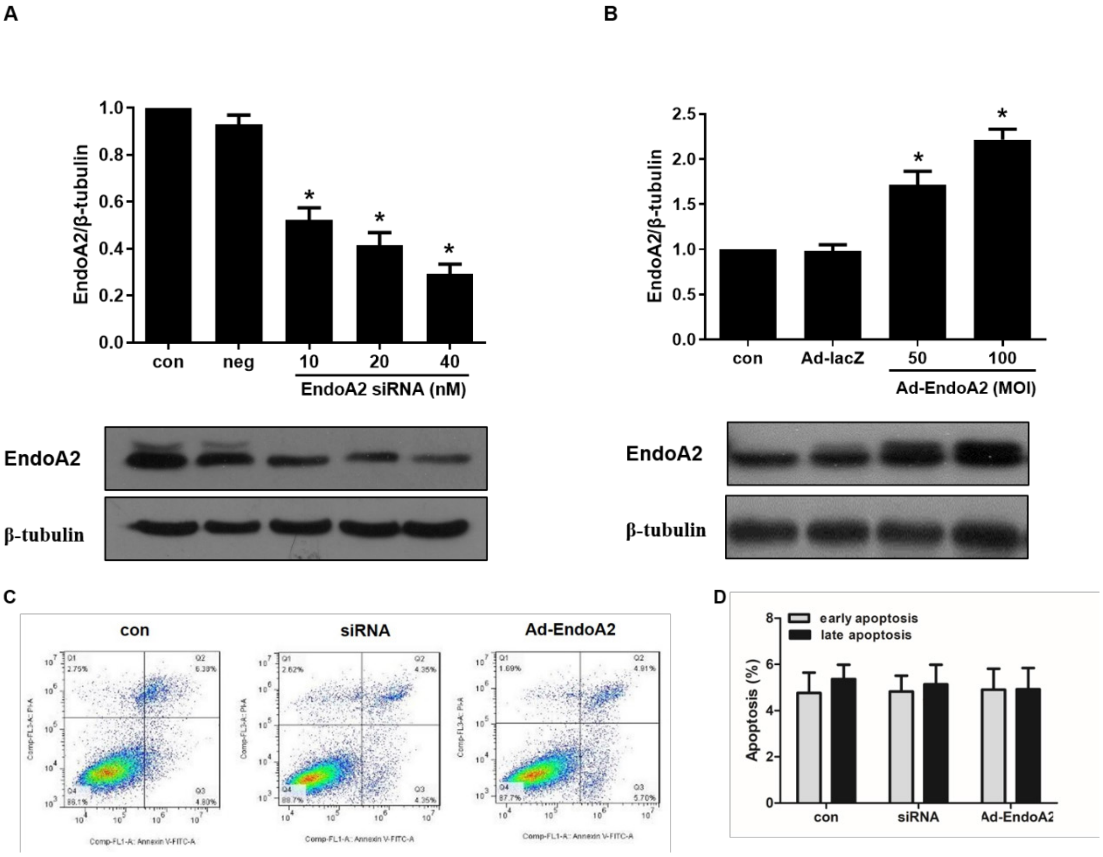

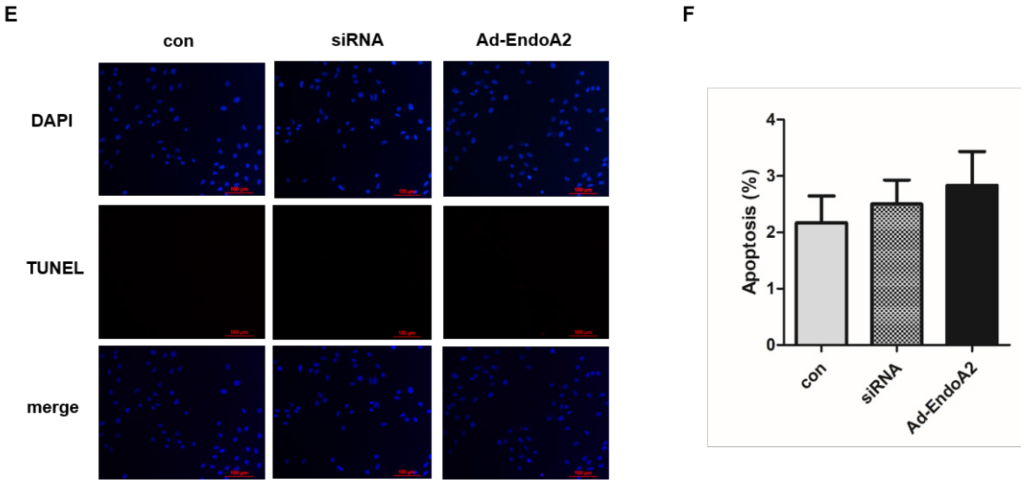

Figure S2. Knockdown or overexpression of EndoA2 had no effect on cell apoptosis. (A) Knockdown efficacy of EndoA2 siRNA in NRCMs. According to our previous studies, we chose to transfect with 20 nM EndoA2-siRNA for 48 h in the following experiment (n=4, $p<0.05$ vs con). **(B)** Overexpression efficacy of Ad-EndoA2 in NRCMs. According to our previous studies, we chose to transfect with 50 MOI Ad-EndoA2 for 48 h in the following experiment (n=4, $p<0.05$ vs con). **(C-D)** Annexin V-FITC/PI flow cytometry analyses showed that knockdown or overexpression of EndoA2 had no effect on cell apoptosis (n=6). **(E-F)** TUNEL and DAPI double-staining showed that knockdown or overexpression of EndoA2 had no effect on cell apoptosis (n=6).

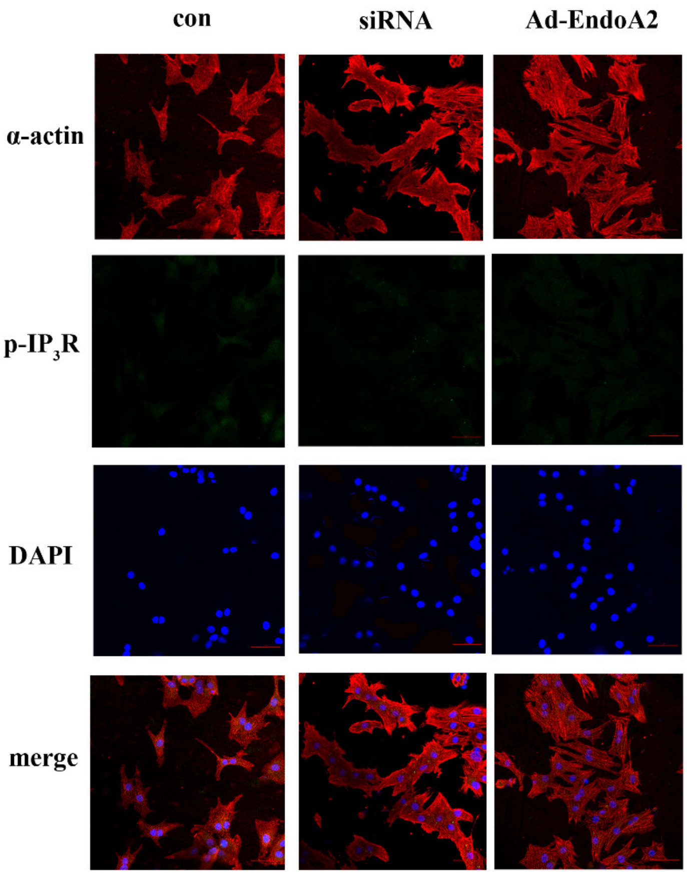

Figure S3. Representative images showed that knockdown or overexpression of EndoA2 had no effect on the phosphorylation of IP_3R. Scale bars=50 μm (n=6).

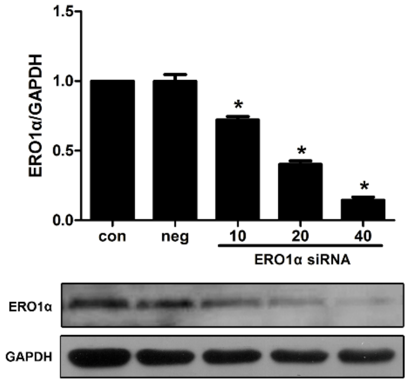

Figure S4. Representative images and densitometric analyses showed that 10-40 nM ERO1α siRNA decreased endogenous ERO1α expression (n=5, *p<0.05 vs. control).

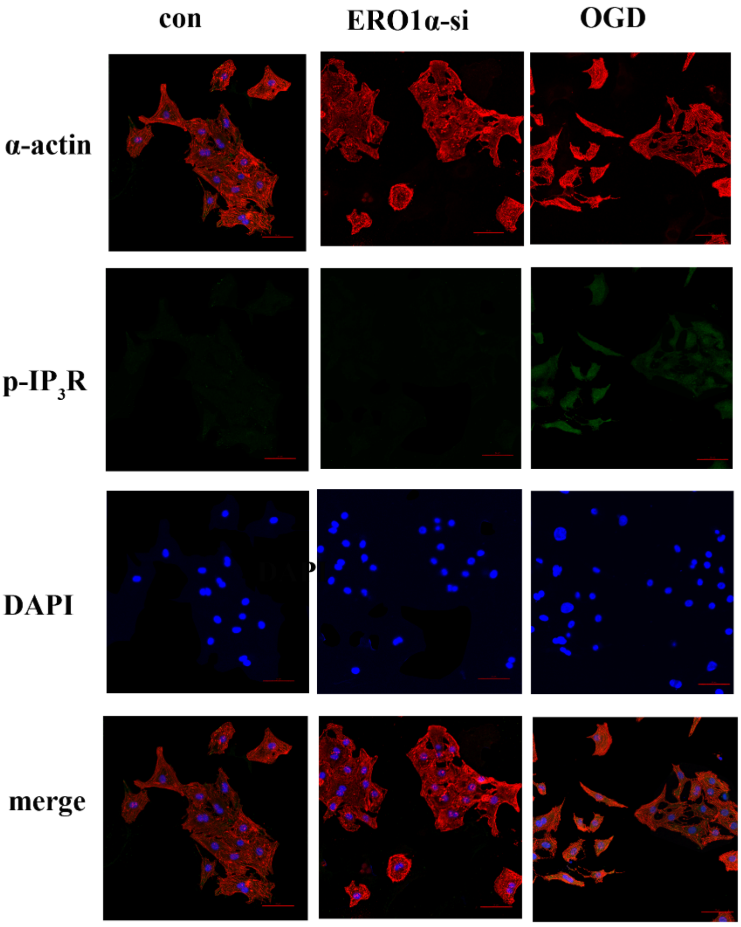

Figure S5. Representative images showed that knockdown of ERO1α had no effect on the phosphorylation level of IP_3R. Scale bars=50 μm (n=6).
